# Supplementary figures and images for: Gain control with A-type potassium current: IA as a switch between divisive and subtractive inhibition
Source: PLoS Comput Biol. 2018 Jul 9;14(7):e1006292. doi: 10.1371/journal.pcbi.1006292 (PMC6053252; doi:10.1371/journal.pcbi.1006292)

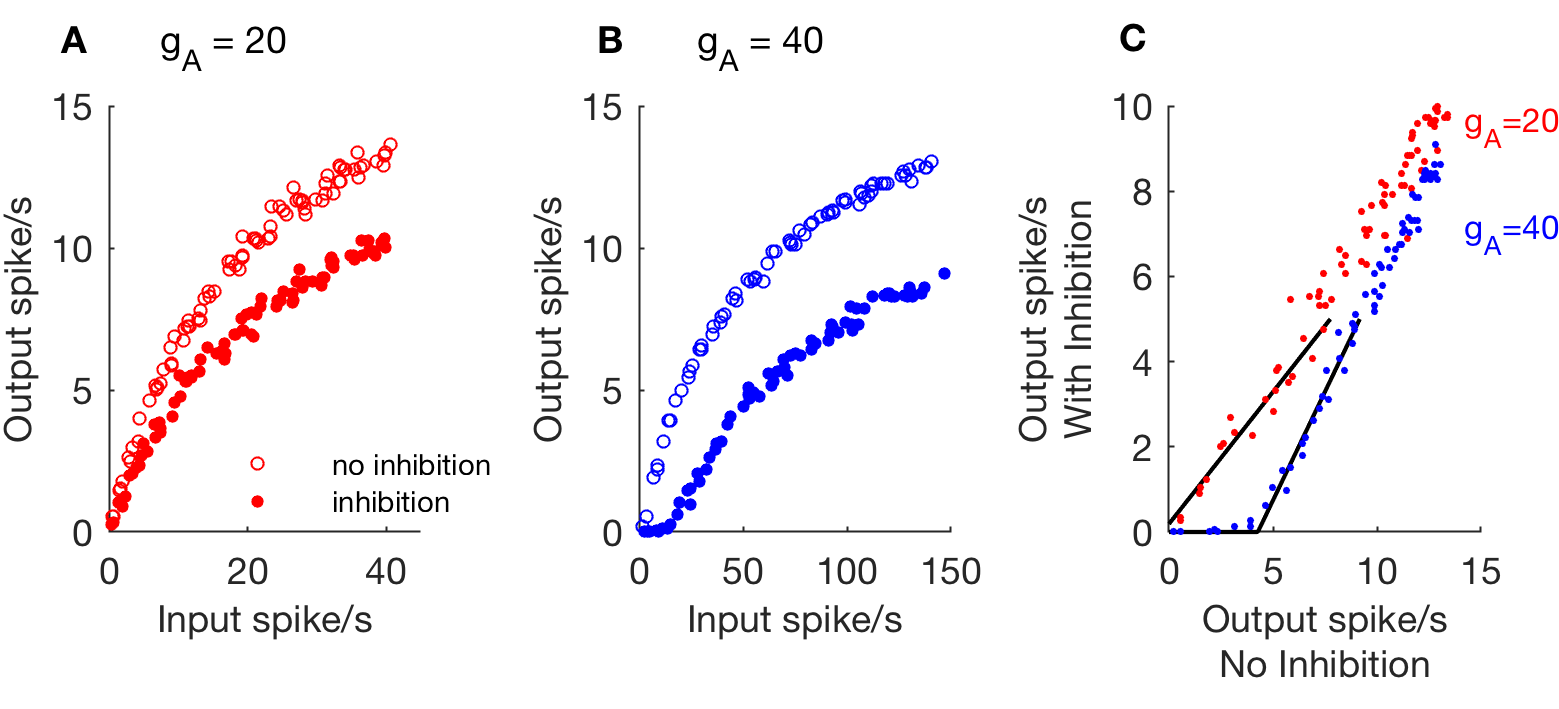

Supplement: S1 Fig — A, B: Output firing rates as a function of excitatory input rate, computed from simulations without inhibition (empty circles, gSyn,I = 0) and with inhibition (filled circles, gSyn,I = 1 and rI = 50 Hz). Excitatory synaptic strength is gSyn,E = 0.5. In A: Divisive rescaling of the input/output relation with gA = 20. In B: Subtractive shifting of the input/output relation with gA = 40. C: Data from A and B are replotted with output firing rates in the absence of inhibition on the ordinate and output firing rates in the presence of inhibition on the abscissa. Threshold-linear functions are fit to simulation data (black lines). Rightward shift of threshold-linear function for gA = 40 is characteristic of subtractive inhibition. (TIF) [file pcbi.1006292.s001.tif]

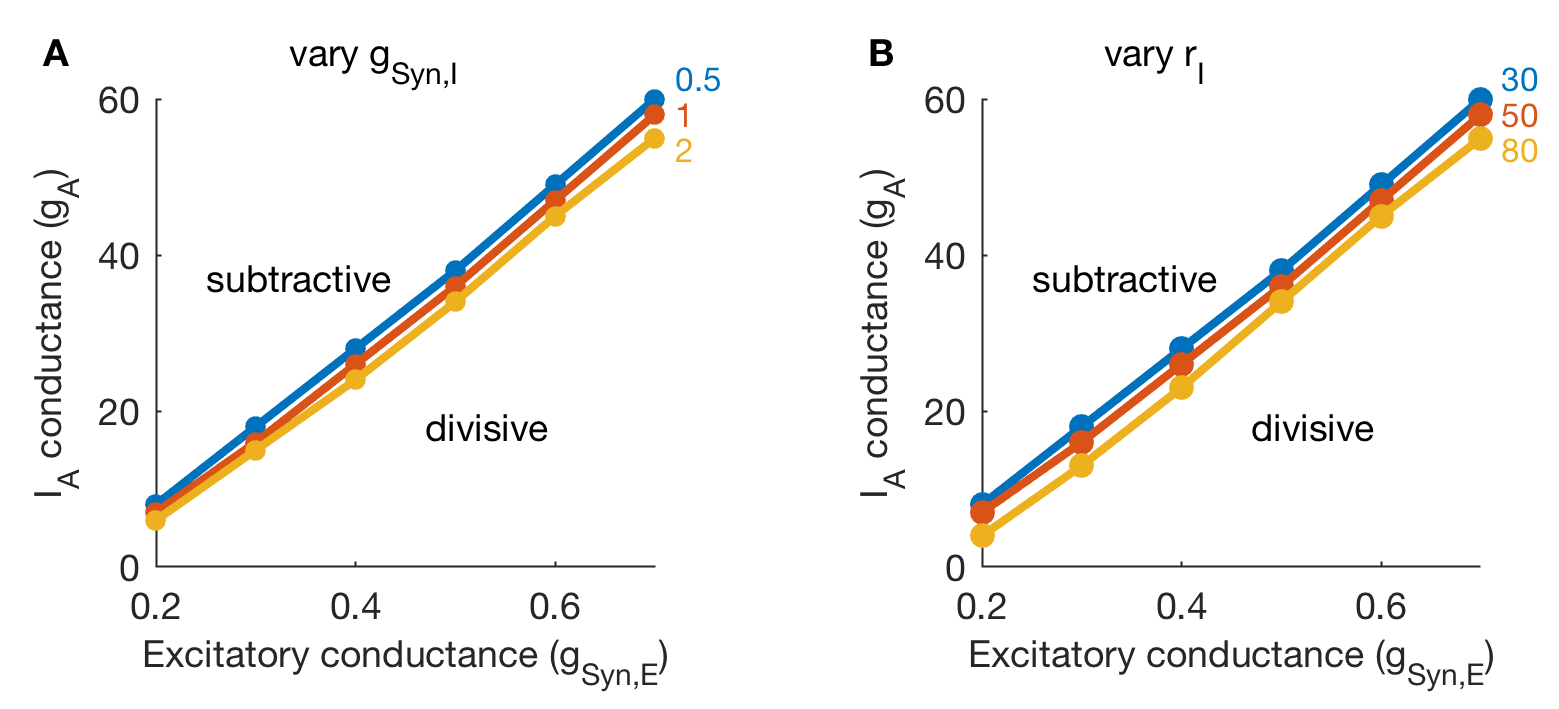

Supplement: S2 Fig — A, B: For each parameter set, we fit threshold-linear functions to characterize the relationship between output firing rates in the presence and absence of inhibition. Dots in each panel identify the smallest value of gA (for a given parameter set) at which inhibition is subtractive. In A: We vary inhibition strength (gSyn,I = 0.5, 1, 2) and keep inhibition rate fixed at 50 Hz. In B: We vary inhibition rate (rI = 30, 50, 70 Hz) and keep inhibition strength fixed at gSyn,I = 1. The values of gA that define the boundary between subtractive and divisive inhibition decrease with increases in either inhibition parameter (gSyn,I or rI). (TIF) [file pcbi.1006292.s002.tif]

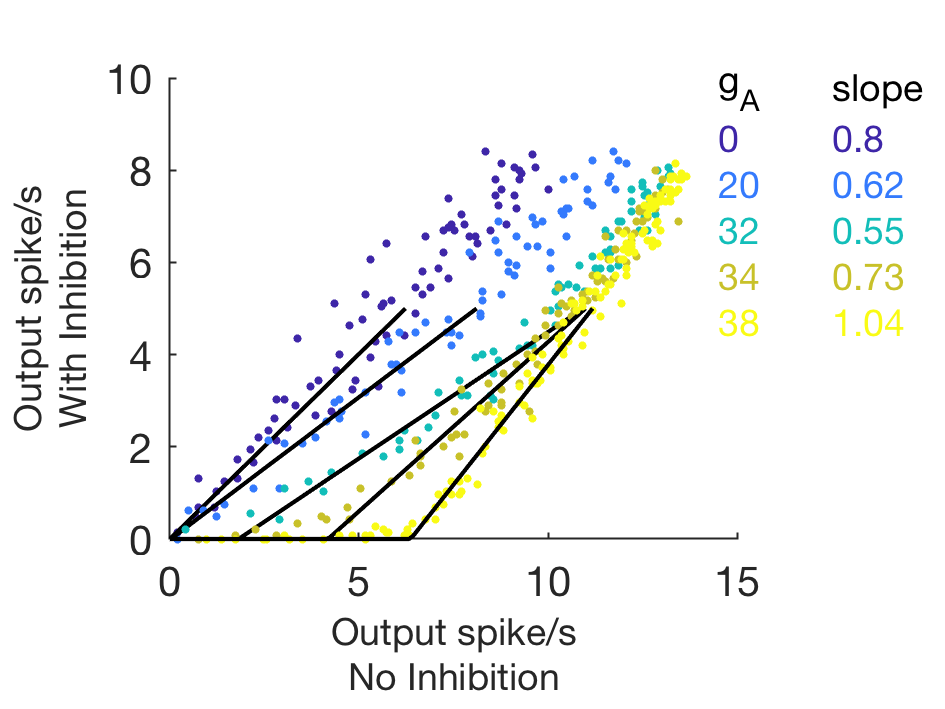

Supplement: S3 Fig — Inpout/output firing rate relations for varying A-channel conductance. Inhibition is divisive for lower gA (compare slopes for gA = 0 and gA = 20), and shows both divisive and subtractive features for higher values of gA (notice rightward-shift of input/output curves, but also changes in slopes indicated in legend). We classify as subtractive any response for which the input/output curve is shifted rightward. Thus, subtractive responses (in our classification) also include “mixed” responses such as those shown here. (TIF) [file pcbi.1006292.s003.tif]

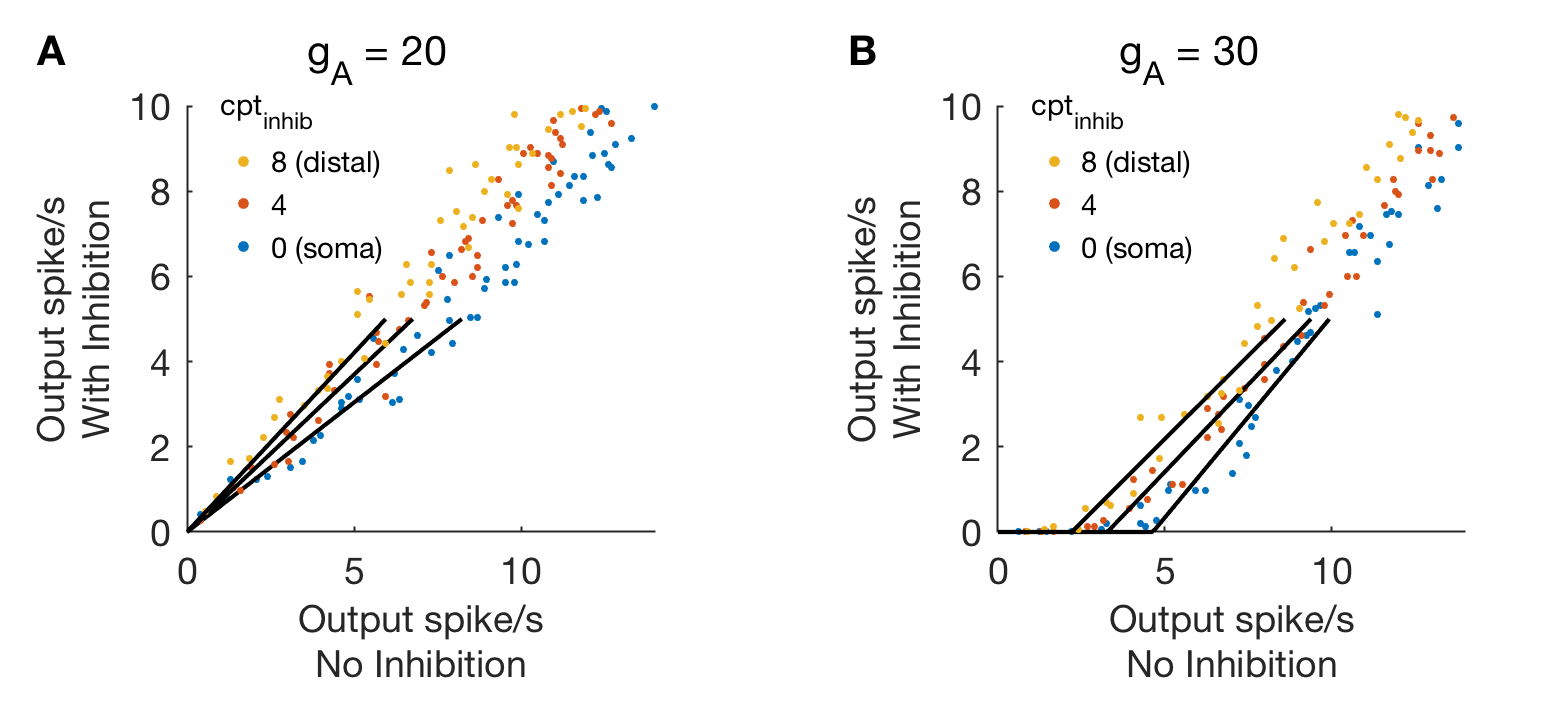

Supplement: S4 Fig — A: Threshold-linear relations between output firing rates in simulations of the multi-compartment model with and without inhibition for varying inhibition input location and gA = 20. For simulations with inhibition: gSyn,I = 1 and rI = 50. Inhibition is divisive for inhibitory inputs that target the soma (cptinhib = 0), an intermediate position on the dendrite (cptinhib = 4, same as locatioin of excitatory input), and a distal location on the dendrite (cptinhib = 8). B: Threshold-linear relations for varying inhibition input location and gA = 30. For simulations with inhibition: gSyn,I = 1 and rI = 50. Inhibition is subtractive for inhibitory inputs that target the soma or positions on the dendrite. (TIF) [file pcbi.1006292.s004.tif]
